# Quantitative evaluation of myocardial layer-specific strain using two-dimensional speckle tracking echocardiography in septic patients

**DOI:** 10.1186/s12871-023-02186-x

**Published:** 2023-08-11

**Authors:** Jin Zhang, Jing Zhu, Tian Xie, Fan Sun, Ni Wang, Feng-Mei Guo

**Affiliations:** 1 Jiangsu Provincial Key Laboratory of Critical Care Medicine, Department of Critical Care Medicine, Zhongda Hospital, School of Medicine, Southeast University, Nanjing, 210009 China; 2 Department of Cardiology, The Second Affiliated Hospital of Soochow University, Suzhou, 215000 China

**Keywords:** Sepsis, GLS, Layer-specific strain, Left ventricular, Sepsis-induced cardiomyopathy

## Abstract

**Background:**

Although global longitudinal strain (GLS) is proven to be reduced and associated with adverse outcomes in septic patients, it has not been elucidated whether or not layer-specific strains are reduced. We aimed to explore the layer-specific strains of left ventricular (LV) for assessing myocardial dysfunction in septic patients.

**Methods:**

A prospective observational study of patients with sepsis was conducted in a tertiary hospital in China. Routine two-dimensional speckle tracking echocardiography was performed within 24 h of enrollment. Demographic data, laboratory values, and clinical outcomes were collected.

**Results:**

We recruited 79 septic patients finally. The mean age of septic patients was 59.4 years old and 45 (57.0%) were male. The median Acute Physiology Age and Chronic Health Evaluation (APACHE II) score, and mean sequential organ failure assessment (SOFA) score of all patients were 19.0 and 7.7, respectively. According to the left ventricular ejection fraction (LVEF) value of 50%, the patients were categorized into two groups: SICM (sepsis-induced cardiomyopathy, LVEF < 50%, n = 22) and non-SICM group ( LVEF ≥ 50%, n = 57). The median LVEF of SICM and non-SICM patients were 41.9% and 58.7%, and SICM patients had less negative layer-specific strain and global strain than that of non-SICM patients. The echocardiographic comparison of non-SICM and healthy controls was conducted to explore the myocardial injuries of non-SICM patients and the non-SICM had worse LS-epi than that of controls (-18.5% vs. -21.4%, *p* = 0.024).

**Conclusion:**

There were 72.2% (57) septic patients presented with non-SICM (LVEF ≥ 50%), and the strain value of epicardium of them was less negative than healthy controls.

## Introduction

Sepsis is a life-threatening infection that could lead to multiple organ dysfunction and is the most common cause of death in critically ill patients [1]. Cardiovascular failure is the most severe manifestation of sepsis, which involves vasoplegia and myocardial dysfunction [2]. Sepsis-induced cardiomyopathy (SICM) occurs in 40-50% of patients with sepsis and significantly increases their mortality of them, although a proportion of patients do not exhibit any clinical signs or symptoms [3]. Left ventricular systolic dysfunction (LVSD) caused by sepsis is particularly common and has been the focus of research in recent years, however, the diagnosis of LVSD in patients with sepsis remains challenging [4].

Echocardiography is currently suggested for evaluating myocardial dysfunction in sepsis and septic shock [5]. Two-dimensional speckle-tracking echocardiography (STE) is a novel technique for the quantitative assessment of myocardial deformation, representing the intrinsic left ventricular (LV function) [6]. Compared with left ventricular ejection fraction (LVEF), LV global longitudinal strain (GLS) measured by STE is more sensitive for evaluating LV systolic function, and it is reported that STE is a predictive marker of mortality for septic patients [7]. Considering the arrangement of myocardial fibers, the LV wall is composed of the endocardium, middle myocardium, and epicardium, and each layer could be damaged with the results of disease severity [8]. The layer-specific strain has been proven to be more sensitive for assessing LV systolic function and may be a critical marker of disease prognosis [9]. Therefore, we conducted a prospective observational study to evaluate the LV dysfunction of each myocardial layer in septic patients using layer-specific strain technique.

## Methods

This was a single-center, prospective observational study at the intensive care unit of a tertiary hospital. The study was approved by the Ethics Committees for Clinical Research of Zhongda Hospital, Southeast University (Approval No: 2022ZDSYLL075-P01).

***Study population*** Adult patients diagnosed with sepsis according to the sepsis-3 definition [1], admitted to intensive care unit of Zhongda Hospital, Southeast University were screened for eligibility from July 15, 2022 to December 31, 2022. Exclusion criteria included: (1) patients with a history of cardiac history and/or other diseases or treatments that affect cardiac function, including coronary heart disease, myocarditis, cardiomyopathy, moderate or severe valvular disease, arrhythmias, post-cardiopulmonary resuscitation status, severe chronic kidney disease, pulmonary thromboembolism and chronic obstructive pulmonary disease, malignant tumor with radiotherapy and chemotherapy, receiving extracorporeal membrane oxygenation; (2) insufficient quality of views; (3) rapid clinical changes do not allow completion of echocardiography; (4) pregnant patients; (5) unable to obtain informed consent. Full clinical information of patients was recorded, including demographics and comorbidities, Acute Physiology Age and Chronic Health Evaluation (APACHE II) score, sequential organ failure assessment (SOFA) score, the etiologies of sepsis, hemodynamics, main laboratory results, organs dysfunction and clinical outcome at 28 days.

***Echocardiography measurements and analysis*** Routine two-dimensional echocardiography was performed by ultrasound (Philips EPIQ 7 C system, Washington, USA) within 24 h of patients’ being admitted to ICU. All echocardiographies were performed by trained operators and views of three consecutive cardiac cycles were recorded using an S5-1 phased array transducer. All measurements were based on guidelines [10]. The size of chambers and thickness of wall were measured firstly, including left ventricular end diastolic diameter (LVEDD), left ventricular end systolic diameter (LVESD), left atrial anterior and posterior diameter (LAAPD), interventricular septal thickness (IVST), posterior wall thickness (PWT). Then LVEDD, LVESD, and LAAPD were corrected by body surface area (BSA), and the relative wall thickness (RWT, 2PWT/LVEDD) was calculated. We recorded the early (E) and late (A) peak velocities of mitral inflow and the E/A ratio. Pulsed-wave tissue Doppler imaging (TDI) was used to assess the early diastolic (e′) velocities, then we took the average of e′ to calculate E/e′. Left ventricular ejection fraction (LVEF) was measured with the biplane method of disk summation according to modified Simpson’s rule. And patients with LVEF < 50% were considered to be SICM [11].

**Fig. 1:**
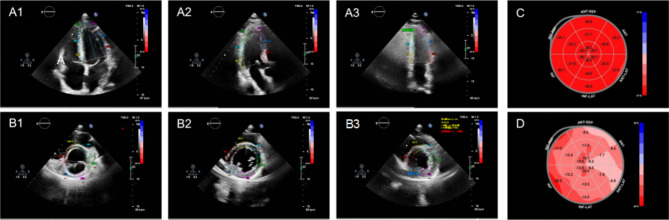
Analysis of layer-specific longitudinal and circumferential strain. The layer-specific longitudinal strain was derived from the apical 4-chamber view (**A1**), 3-chamber view (**A2**), and 2-chamber view (**A3**). The layer-specific circumferential strain was derived from the basal short-axis view (**B1**), midventricular short-axis view (**B2**), and apical short-axis view (**B3**). (**C**) Epicardial myocardial circumferential strain of a health. (**D**) Epicardial myocardial circumferential strain of a septic patient

***Layer-Specific Strain Analysis*** The obtained images were digitally transmitted to Qlab aCMQ Package (Philips EPIQ 7 C system, Washington, USA) for offline analysis, which tracked endocardial and epicardial borders on the apical 2-chamber, apical 3-chamber, apical 4-chamber views, and parasternal short-axis views at three levels (basal, midventricular, and apical). The software algorithm could generate automatically the strain on every view and the global strain of each layer. The longitudinal strain of three layers (LS-endo, LS-mid, LS-epi) were analyzed in three apical views, and the circumferential strain of three layers (CS-endo, CS-mid, CS-epi) in the parasternal short-axis views at three levels (basal, midventricular, and apical), as shown in Fig. 1.

***Statistical analysis*** Statistical analysis was performed using SPSS software (version 23.0, IBM SPSS Inc., Chicago, IL, USA). Continuous variables were reported as the mean and standard deviation, or median and interquartile range depending on the distribution. Dichotomous variables were reported as numbers and percentages. Student′s t tests and nonparametric tests were used to compare the continuous data between the two groups and Chi-square tests was used to compare dichotomous data. Spearman′s correlation was used to determine the association of strain values and clinical parameters.

## Results

**Fig. 2:**
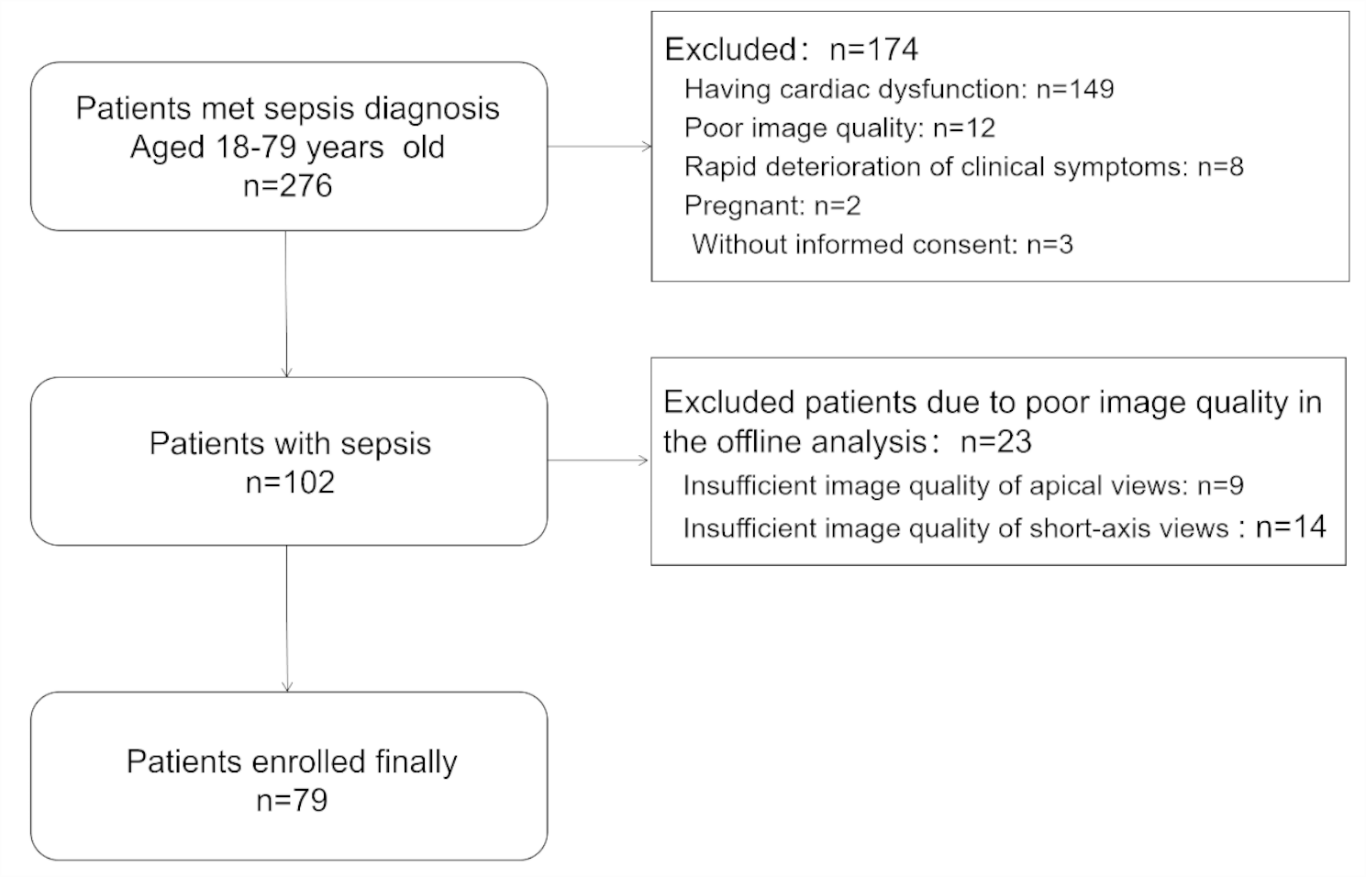
Flow Chart

***Recruitment*** A total of 276 septic patients were admitted to the intensive care unit consecutively and screened for eligibility during the study period. And 79 patients were included in the final analysis. Of enrolled patients, 149 patients were excluded due to cardiac disease or other disease or treatment affecting cardiac function; 12 patients were excluded because of poor image quality and 23 patients were excluded due to the next offline analysis, detailed information was given in Fig. 2.

***Patient characteristics*** Baseline characteristics of all patients in the study were provided in Table 1. The mean age was 59.4 years, and 57.0% of them were male. The median APACHE II score and mean SOFA score of all patients were 19.0 and 7.7, respectively. Among 79 patients, 60 (76.0%) were receiving mechanical ventilation at the time of echocardiography, and 54 (68.4%) had septic shock. The responsible etiologies of sepsis were respiratory infection (31.6%), abdominal infection (45.6%), urinary infection (13.9%), and other (12.7%). 21 (26.6%) patients died within 28 days. There was no difference in baseline data between the SICM and non-SICM except that the non-SICM patients had older age (62.5 years vs. 53.5 years, *p* = 0.011) and lower NT-pro BNP value (1590.0 pg/ml vs. 6055.0 pg/ml, *p* = 0.040) than SICM patients.

**Table 1:** Baseline characteristics of septic patients admitted to intensive care unit

	All (n = 79)	SICM (LVEF < 50%) (n = 22)	non-SICM (LVEF ≥ 50%) (n = 57)	*p*
*Demographics and comorbidities*				
Age (years)	59.4 ± 13.6	53.5 ± 16.6	62.5 ± 12.6	0.011
Male, n (%)	45 (57.0%)	11 (50%)	34 (%)	0.459
Body weight (kg)	66.4 ± 11.2	66.4 ± 12.0	66.3 ± 11.0	0.913
Hypertension, n (%)	36 (45.6%)	10 (45.5%)	26 (%59.6)	0.608
Diabetes, n (%)	28 (35.4%)	4 (18.2%)	24 (42.1%)	0.169
Cerebrovascular disease, n (%)	22 (27.8%)	5 (22.7%)	17 (29.8%)	1.000
APACHE II score^*^	19.0 [12.0–25.0]	14.5 [10.8–19.3]	19.0 [13.5–25.0]	0.051
SOFA score	7.7 ± 4.1	6.5 ± 3.7	8.1 ± 4.2	0.121
*Source of infection*				
Respiratory, n (%)	25 (31.6%)	4 (18.2%)	21 (36.8%)	0.176
Abdominal, n (%)	36 (45.6%)	11 (50%)	25 (43.9%)	0.801
Urinary, n (%)	11 (13.9%)	3 (13.6%)	8 (14.0%)	1.000
Others, n (%)	10 (12.7%)	4 (18.2%)	6 (10.5%)	0.452
*Hemodynamics*				
MAP (mmHg)	85.5 ± 14.1	84.0 ± 16.7	86.4 ± 13.1	0.509
Heart rate (beats per minute)	92.3 ± 18.6	102.0 ± 14.7	95.9 ± 16.7	0.142
CVP (mmHg)^*^	8.0 [6.0–11.0]	8.5 [6.5–11.0]	8.0 [6.5–10.5]	0.783
*Organ failures*				
Mechanical ventilation, n (%)	60 (76.0%)	17 (77.3%)	43 (75.4%)	1.000
Renal replacement therapy, n (%)	12 (15.2%)	5 (22.7%)	7 (12.3%)	0.307
Septic shock, n (%)	54 (68.4%)	18 (81.8%)	36 (63.2%)	0.176
*Laboratory results*				0.236
Arterial blood lactates (mmol/L)^*^	2.5 [1.4–4.6]	3.1 [1.4–5.9]	2.3 [1.3–3.6]	0.225
Troponin I (ng/ml)^*^	0.02 [0.01–0.08]	0.02 [0.01–0.17]	0.02 [0.01–0.05]	0.405
NT-proBNP (pg/ml)^*^	1830.0 [411.0-7890.0]	6055.0 [421.0-22425.0]	1590.0 [389.5–4292.0]	0.040
*Clinical outcomes*				
Death within 28 days, n (%)	21 (26.6%)	7 (31.8%)	14 (24.6%)	0.574
Length of ICU stay (days)^*^	6.1 [2.3, 15.2]	10.6 [2.3, 15.6]	5.8 [2.3, 12.7]	0.391
Length of hospital stay (days)^*^	17.5 [7.8, 26.0]	16.5 [11.8, 23.3]	18.0 [7.0, 27.5]	0.523

Data are expressed as mean ± SD or number (%) unless stated otherwise, *MAP* mean arterial pressure, *CVP* central venous pressure, *NT-proBNP* N-terminal pro-B type natriuretic peptide, *APACHE* Acute Physiology Age and Chronic Health Evaluation, *SOFA* sequential organ failure assessment

^*^Median (interquartile range)

***Conventional echocardiographic measurements*and*layer-specific strain analysis*** The median LVEF of SICM and non-SICM patients were 41.9% and 58.7%, respectively. SICM patients had larger LVESD/BSA than non-SICM patients (19.5 mm/m^2^ vs. 18.2 mm/m^2^, *p* = 0.021). There was no difference in other conventional echocardiographic parameters between the two groups. The LS-endo (-14.8% vs. -20.6%, *p* < 0.0001), LS-mid (-14.0% vs. -19.7%, *p* < 0.0001), LS-epi (-13.5% vs. -18.5%, *p* < 0.0001) and GLS (-14.0% vs. -19.6%, *p* < 0.0001) in SICM were significantly less negative than that of the non-SICM group. In circumference direction, SICM had worse CS-endo (-23.0% vs. -29.4%, *p* < 0.0001), CS-epi (-17.3% vs. -20.8%, *p* = 0.047), and GCS (-19.7% vs. -24.1%, *p* = 0.007) than non-SICM group, as given in Table 2.

**Table 2:** Conventional echocardiography measurements and layer-specific strain of septic patients admitted to intensive care unit

	All (n = 79)	SICM (LVEF < 50%) (n = 22)	non-SICM (LVEF ≥ 50%) (n = 57)	*p*
***Conventional echocardiography***				
LVEDD/BSA (mm/m^2^)	27.3 ± 4.2	27.6 ± 4.1	27.2 ± 4.3	0.696
LVESD/BSA (mm/m^2^)	17.8 ± 3.9	19.5 ± 4.2	18.2 ± 4.2	0.021
LAAPD/BSA (mm/m^2^)	18.0 ± 3.9	17.6 ± 3.1	18.2 ± 4.2	0.572
RWT^*^	0.4 [0.3–0.5]	0.4 [0.3–0.5]	0.4 [0.4–0.5]	0.608
LVEF (%)	54.1 ± 10.1	41.9 ± 5.8	58.7 ± 7.0	<0.0001
E (cm/s)	83.7 ± 20.3	80.5 ± 18.2	84.9 ± 20.8	0.388
A (cm/s)	76.2 ± 22.3	76.3 ± 24.5	76.2 ± 21.6	0.976
E/A^*^	1.1 [0.8–1.4]	1.0 [0.7–1.8]	1.1 [0.9–1.4]	0.484
Average e′ (cm/s)^*^	9.3 [7.8–11.1]	9.6 [7.4–11.3]	9.3 [7.9–11.1]	0.848
E/e′^*^	9.3 [7.2–10.8]	9.3 [6.2–11.0]	9.2 [7.2–10.8]	0.969
***Longitudinal Strain***				
LS-endo (%)	-19.0 ± 5.5	-14.8 ± 3.6	-20.6 ± 5.2	< 0.0001
LS-mid (%)	-18.1 ± 5.2	-14.0 ± 3.3	-19.7 ± 5.0	<0.0001
LS-epi (%)	-17.0 ± 4.9	-13.5 ± 3.1	-18.5 ± 4.7	< 0.0001
GLS (%)	-18.0 ± 5.2	-14.0 ± 3.2	-19.6 ± 4.9	< 0.0001
***Circumferential Strain***				
CS-endo (%)	-27.6 ± 8.0	-23.0 ± 5.9.	-29.4 ± 8.0	<0.0001
CS-mid (%)	-21.3 ± 7.6	-18.9 ± 6.1	-22.2 ± 7.9	0.081
CS-epi (%)	-19.9 ± 7.1	-17.3 ± 5.6	-20.8 ± 7.4	0.047
GCS (%)	-22.9 ± 7.4	-19.7 ± 5.7	-24.1 ± 7.6	0.007

Data are expressed as mean ± SD unless stated otherwise. *LVEDD* left ventricular end diastolic diameter, *LVESD* left ventricular end systolic diameter, *LAAPD* left atrial anterior and posterior diameter, *BSA* body surface area, *RWT* relative wall thickness, *LVEF* left ventricular ejection fraction, *E* mitral inflow peak early diastolic velocity (pulsed doppler), *A* mitral inflow peak late diastolic velocity (pulsed doppler), *Average e*′ average of lateral and interventricular mitral diastolic velocities (tissue doppler imaging). *LS* global longitudinal strain, *CS* global circumferential strain, *endo* endocardium, *mid* middle myocardium, *epi* epicardium. *GLS* global longitudinal strain, *GCS* global circumferential strain, *endo* endocardium, *mid* middle myocardium, *epi* epicardium

^*^Median (interquartile range)

**Fig. 3:**
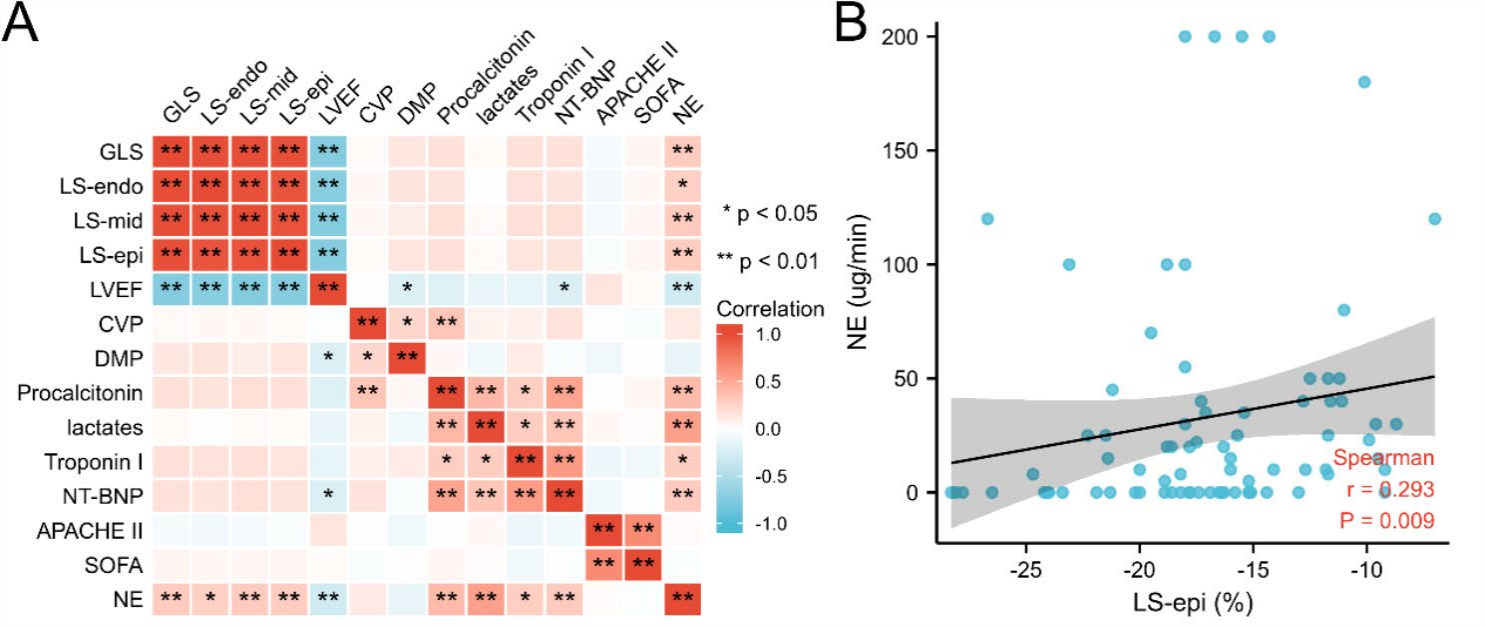
Correlation heatmap of strains and clinical parameters. (*GLS* global longitudinal strain, *LS-endo LS-mid LS-epi* Longitudinal strain in the endocardium, middle myocardium, epicardium, *LVEF* left ventricular ejection fraction, *CVP* central venous pressure, *DAP* Diastolic arterial pressure, *NT-proBNP* N-terminal pro-B type natriuretic peptide, *APACHE* Acute Physiology Age and Chronic Health Evaluation, *SOFA* sequential organ failure assessment, *NE* maximal dose of norepinephrine)

As provided in Table 3, the echocardiographic values of non-SICM patients were compared to age and gender-matched healthy volunteers to identify the myocardial injuries of non-SICM patients. There was no difference in conventional echocardiographic values and GLS between the two groups while non-SICM had less negative LS-epi than that of controls (-18.5% vs. -21.4%, *p* = 0.024). The correlation of longitudinal strains and clinical parameters was evaluated in Fig. 3, and there was a significant correlation between strain values and maximal dose of norepinephrine.

**Table 3:** Conventional echocardiography measurements and layer-specific strain of non-SICM and control group

	non-SICM (LVEF ≥ 50%) (n = 57)	Control (n = 21)	*p*
***Conventional echocardiography***			
LVEDD/BSA (mm/m^2^)	27.2 ± 4.3	27.5 ± 3.2	0.763
LVESD/BSA (mm/m^2^)	18.2 ± 4.2	18.4 ± 3.	0.183
LA/BSA (mm/m^2^)	18.2 ± 4.2	17.8 ± 3.1	0.718
RWT^*^	0.4 [0.4–0.5]	0.4 [0.3–0.4]	0.071
LVEF (%)	58.7 ± 7.0	62.0 ± 7.8	0.086
E (cm/s)	84.9 ± 20.8	80.6 ± 13.9	0.382
A (cm/s)	76.2 ± 21.6	73.4 ± 17.5	0.597
E/A^*^	1.1 [0.9–1.4]	1.1 [0.9–1.3]	0.933
Average e′^*^	9.3 [7.9–11.1]	10.1 [8.2–12.4]	0.370
E/e′ (cm/s) ^*^	9.2 [7.2–10.8]	8.0 [6.4–9.6]	0.092
***Longitudinal Strain***			
LS-endo (%)	-20.6 ± 5.2	-22.7 ± 5.0	0.121
LS-mid (%)	-19.7 ± 5.0	-21.9 ± 5.0	0.085
LS-epi (%)	-18.5 ± 4.7	-21.4 ± 5.0	0.024
GLS (%)	-19.6 ± 4.9	-22.0 ± 4.9	0.063
***Circumferential Strain***			
CS-endo (%)	-29.4 ± 8.0	-32.3 ± 8.9	0.177
CS-mid (%)	-22.2 ± 7.9	-25.3 ± 9.2	0.147
CS-epi (%)	-20.8 ± 7.4	-22.9 ± 8.3	0.282
GCS (%)	-24.1 ± 7.6	-26.8 ± 8.3	0.179

Data are expressed as mean ± SD unless stated otherwise. *LVEDD* left ventricular end diastolic diameter, *LVESD* left ventricular end systolic diameter, *LAAPD* left atrial anterior and posterior diameter, *BSA* body surface area, *RWT* relative wall thickness, *LVEF* left ventricular ejection fraction, *E* mitral inflow peak early diastolic velocity (pulsed doppler), *A* mitral inflow peak late diastolic velocity (pulsed doppler), *Average e*′ average of lateral and interventricular mitral diastolic velocities (tissue doppler imaging). *LS* global longitudinal strain, *CS* global circumferential strain, *endo* endocardium, *mid* middle myocardium, *epi* epicardium. *GLS* global longitudinal strain, *GCS* global circumferential strain, *endo* endocardium, *mid* middle myocardium, *epi* epicardium

^*^Median (interquartile range)

## Discussion

The prevalence of cardiac dysfunction is high in patients with sepsis, especially LV dysfunction. The classic evaluation for the diagnosis of LV function depends on LVEF, neglecting early myocardial damage in septic patients and the inconsistency between LVEF and outcomes of septic patients [12]. There is a lack of enough recognition of sepsis-induced myocardial dysfunction [13]. Here we explored the conventional echocardiography parameters and layer-specific strain in septic patients. The highlights of our study are as follows. First, the longitudinal and circumferential strains of each myocardial layer are reduced not only GLS. More importantly, we found a reduction in the longitudinal strain of epicardium in non-SICM patients compared with the healthy controls while conventional echocardiography parameters and GLS were comparable between the two groups.

We demonstrated that 22 (27.8%) septic patients presented with SICM (LVEF ≥ 50%), and 28-day mortality of them was 31.8%, which was higher than that of non-SICM patients. Although the difference was not significant, the result was inconsistent to new recognition of worse prognosis in hyperdynamic septic patients [12]. The probable cause is that we excluded strictly any patients suspected of cardiac insufficiency.

New focuses have shown the superiority of two-dimensional speckle tracking in assessing LV systolic function in septic patients [14]. Compared with LVEF, GLS reflected myocardial dysfunction more accurately in septic patients and was associated with poor prognosis [15]. In our study, the SICM group showed lower GLS and layer-specific strain than that of non-SICM, indicating that SICM patients had more myocardial damage. And we attempted to assess cardiac function in non-SICM patients. No differences in conventional echocardiography parameters and GLS between non-SICM patients and controls in our study. These reflected critically and easily ignored attention in the subgroup of septic patients with higher LVEF. However, layer-specific strain exhibited its superiority and we found the longitudinal strain of epicardium decreased in non-SICM patients. The phenomenon for these was not understood.

Intense immune responses cause inflammatory mediators and vasoactive substances to produce and release in sepsis that could act sensitively on the proximal coronary artery, which could cause spasm of the proximal coronary artery and endothelial injury, and result in damage to the epicardium of dogs [16]. It was reported that myocardial depression of septic patients was stress cardiomyopathy [17], and it has been proved that the increase of catecholamines or other stress-related neuroreceptors leads to spasm of epicardial multivessel spasm in stress cardiomyopathy [18, 19]. These may contribute to the potential mechanisms of reduced strains in the middle myocardium and epicardium in septic patients. A decrement of strains in the middle myocardium and epicardium may be associated with preserved LVEF. Due to the different radius of myocardial layers, the innermost myocardium is passively contracted by the interaction of another myocardium to maintain the LVEF. When the outermost myocardium contracts, its radius shortens as the thickness increases, resulting in the inner myocardium shortening to increase LVEF [20]. Further studies are needed for mechanisms of sepsis-induced myocardial dysfunction.

The correlation between strain values and clinical parameters was explored in our study. Longitudinal strain and maximal dose of norepinephrine have a significant positive correlation, suggesting that poor left ventricular systolic function may be related to higher afterload.

### Limitations

This study has some limitations. Firstly, this study is a single-center study with small sample size and lack of evidence of external verification. And studies in recent years proved the importance of left atrial function for septic patients and the prognosis of patients with severe sepsis is related to right ventricular dysfunction [21, 22]. However, this study focuses on the change of left ventricular in patients with sepsis, and our team will devote to this research in the future.

## Conclusion

Although septic patients with higher LVEF were recognized to be non-SICM, layer-specific strain can detect myocardial damage that could not be diagnosed by conventional ultrasound or GLS in non-SICM patients. The clinical significance of this finding remains to be determined and whether a reduction of longitudinal strain of epicardium in non-SICM patients is associated with long-term poorer prognosis, should be investigated in future prospective studies.

## Data Availability

The datasets used and/or zanalyzed during the current study are available from the corresponding author upon reasonable request.

## Abbreviations

SICM: Sepsis-induced cardiomyopathy
LVSD: Left ventricular systolic dysfunction
STE: Speckle-tracking echocardiography
LV: Left ventricular
LVEF: Left ventricular ejection fraction
GLS: Global longitudinal strain
APACHE II: Acute Physiology Age and Chronic Health Evaluation
SOFA: Sequential organ failure assessment
LVEDD: Left ventricular end diastolic diameter,
LVESD: Left ventricular end systolic diameter,
LAAPD: left atrial anterior and posterior diameter
BSA: Body surface area,
IVST: Interventricular septal thickness,
PWT: Posterior wall thickness,
RWT: Relative wall thickness,
E: Mitral inflow peak early diastolic velocity (pulsed doppler),
A: Mitral inflow peak late diastolic velocity (pulsed doppler)
TDI: Tissue doppler imaging
e′: Early diastolic velocities
LS-endo: Longitudinal strain of endocardium
LS-mid: Longitudinal strain of middlewall
LS-epi: Longitudinal strain of epicardium
CS-endo: Circumferential strain of endocardium
CS-mid: Circumferential strain of middlewall
CS-epi: Circumferential strain of epicardium
GCS: Global circumferential strain
NT-proBNP: N-terminal pro-B type natriuretic peptide
